# Tracking the tumor invasion front using long-term fluidic tumoroid culture

**DOI:** 10.1038/s41598-017-10874-1

**Published:** 2017-09-07

**Authors:** Koh Meng Aw Yong, Zida Li, Sofia D. Merajver, Jianping Fu

**Affiliations:** 1 Department of Mechanical Engineering, University of Michigan, Ann Arbor, MI 48109 USA; 2 Department of Biomedical Engineering, University of Michigan, Ann Arbor, MI 48109 USA; 3 Department of Internal Hematology and Oncology, University of Michigan Medical School, Ann Arbor, MI 48109 USA; 4 Department of Cell and Developmental Biology, University of Michigan Medical School, Ann Arbor, MI 48109 USA; 5 Present Address: Department of Internal Medicine, Hematology, and Oncology, University of Michigan Medical School, Ann Arbor, MI 48109 USA

## Abstract

The analysis of invading leader cells at the tumor invasion front is of significant interest as these cells may possess a coordinated functional and molecular phenotype which can be targeted for therapy. However, such analyses are currently limited by available technologies. Here, we report a fluidic device for long-term three-dimensional tumoroid culture which recapitulated the tumor invasion front, allowing for both quantification of invasive potential and molecular characterization of invasive leader cells. Preliminary analysis of the invasion front indicated an association with cell proliferation and higher expression of growth differentiation factor 15 (GDF15). This device makes real-time tracking of invading leader cell phenotypes possible and has potential for use with patient material for clinical risk stratification and personalized medicine.

## Introduction

Analysis and characterization of invasive subpopulations of cancer cells such as leader cells at the tumor invasion front is an area of interest for cancer research, as cancer cells located at the invasion front possess invasive phenotypes and characterizing these cells may help identify molecular targets for treating tumors no longer organ confined. However, despite their clinical importance, spatiotemporal analysis of leader cells at the tumor invasion front remains a significant challenge^1, 2^.

Currently, studying tumor behavior such as invasion using three-dimensional (3D) models is considered as a better approach compared to two-dimensional (2D) methods^3–5^. Such 3D tumor models include patient derived xenograft (PDX) tumors using immunocompromised animal models such as mice, organoid cultures, and microfluidics^6–8^. However, studying dynamic tumor behaviors using intra-vital imaging in animal models requires specialized equipment and trained personnel. While organoid culture is easy to perform and can recapitulate the tumor invasion front with the potential to be used for drug screening^3, 9, 10^, maintaining organoid culture often requires disturbing the culture, which complicates long-term tracking of tumor invasion into the surrounding hydrogel^5, 8^. A typical organoid culture contains different sized organoids randomly arranged in a dish. Such variations in the initial spatial arrangement and sizes of organoids can confound tracking of cell invasion. Organoid culture is usually not exposed to interstitial flow or pressure, two key forces experienced by invading cancer cells *in vivo* brought about by blood or lymphatic flow^11, 12^. These forces have been demonstrated to play a role in invasion and including them in such studies may shed insight into the biology of cancer invasion^13–17^. In addition, organoid culture starts off with either a single cell or allowing thousands of cells to self-aggregate^18^. It is uncertain whether either approach can fully capture the tumor heterogeneity in a patient tumor which is larger and contains more cells than that of organoids^5^. Thus, rare subpopulations of cancer cells responsible for invasion or chemoresistance may be missed when using organoids to study invasion. Microfluidics holds the potential both for tracking tumor invasion at the single cell level as well as for high-throughput drug screening. However, most existing microfluidic devices are unsuitable for long-term cell culture, and they cannot recapitulate the *in situ* histology seen in organoid or animal models due to their small device size. Furthermore, most microfluidic devices utilized for studying invasion focus on the invasive profile of a collection of single cells rather than cells originating from the invasion front of a tumor mass or organoid grown in a 3D hydrogel matrix^19–22^. While there has been a recent increase in engineered microfluidic devices that attempt to study 3D invasion from a tumor mass, these devices have not been shown to achieve long-term culture^23–27^.

To address these limitations of conventional organoid and microfluidic culture, we developed a fluidic device to recreate a tumor mass (tumoroid) by first localizing a large starting number of PC3 or DU145 prostate cancer cells (1 × 10^6^) to a specific starting position and size (500 μm) using a molded fluidic channel embedded in a collagen hydrogel. A starting diameter of 500 μm was chosen as it has been shown that spheroids of that size recapitulate features of solid tumors such as hypoxia or necrosis in the tumor center^28^. The device further introduces a fluid flow through a separate channel parallel to the tumoroid which serves to provide a steady source of nutrient as well as interstitial pressure and flow. The device is stable up to at least three weeks in culture under such conditions and allowed us to perform spatiotemporal analysis of leader cells at the tumor invasion front.

## Results

### Recapitulation of the tumor invasion front enables for quantification of invasive potential over time

**Figure 1:**
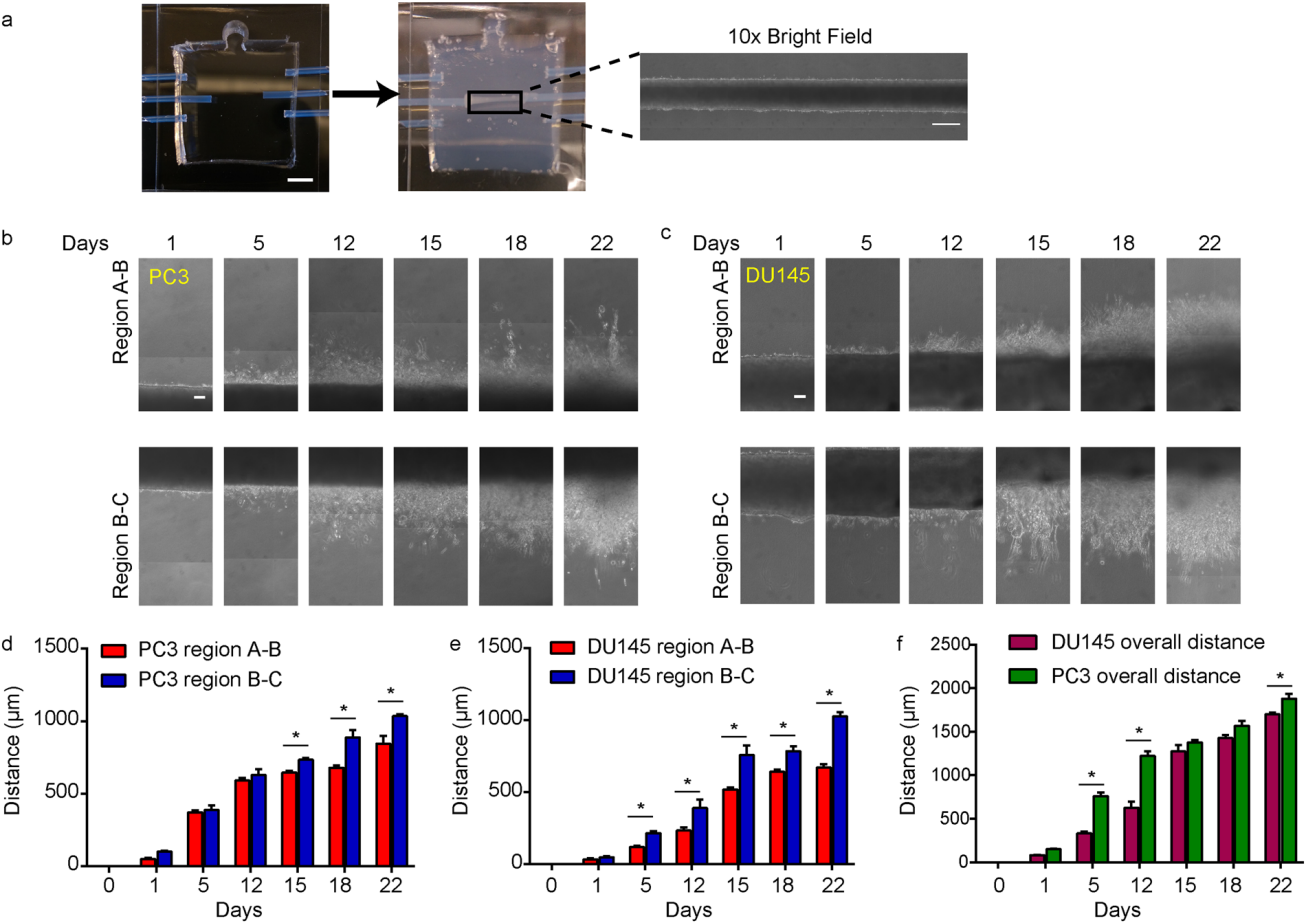
Long-term tumoroid culture recapitulates the invasion front from a tumor mass. (**a**) Overview of tumoroid culture. Syringe needles were placed in an empty device (left) through three channel openings; scale bar = 5mm. Fluidic channels were formed after injection and polymerization of 1 mg/mlcollagen gel (middle). Cancer cells were then introduced into the middle channel before both ends of the channel was sealed to form a tumoroid (right); scale bar = 500 μm. (**b** and **c**) Formation of invasion front in PC3 (**b**) and DU145 (**c**) tumoroids over 3 weeks. Scale bars = 100 μm. (**d** and **e**) Quantification of average invasion distance on both sides of PC3 (**d**) and DU145 (**e**) tumoroids measured over 3 weeks. (**f**) Comparison of overall invasion of PC3 and DU145 tumoroids over 3 weeks. Data were plotted as the mean ± s.e.m, with *n* = 3 biological replicates. *P*-values for each time point were calculated using two tailed unpaired *t*-test. *P < 0.05.

The fluidic device consisted of three molded fluidic channels (A, B and C) running parallel through a 1 mg/ml collagen I gel (Fig. 1a). Two prostate cancer cells, DU145 and PC3, were seeded into the middle fluidic channel B forming a tumoroid 500 μm in diameter (Fig. 1a and Supplemental Fig. 1). Over a period of 3 weeks, a peristaltic pump was used to drive growth medium flow through channel A at a flow rate of 30 μl min^−1^, while the growth media in channel C was static as a control. Invasion into the surrounding collagen I hydrogel was observed on both sides of channel B (between channels A and B: region A-B; between channels B and C: region B-C) (Fig. 1b,c). PC3 cells were more invasive than DU145 as determined by the maximum invasion distance (defined as the longest distance from a leader cell to channel B) in the first two weeks of culture, consistent with the known invasive potential difference between the two cell lines^29^. However, after three weeks of culture, this difference could only be observed in region A-B; PC3 (800 μm), compared to DU145 (685 μm) (Fig. 1d,e). Interestingly, for both DU145 and PC3, invasion distance into the collagen gel was greater in region B-C than in region A-B at 3 weeks. The difference between invasion distance in region A-B and B-C was likely due to an increased fluid pressure within region B-C, which is known to affect tumor invasion^13, 30^ (Supplementary Fig. 2). In our study, it appeared that an increased fluid pressure would promote tumor invasion into the surrounding extracellular matrix. While PC3 cells were overall two times more invasive than DU145 at 12 days of culture, this difference was rapidly lost by 3 weeks, suggesting that the invasive potential of cells may change over time and highlights the importance of utilizing long-term tumoroid culture to study invasion (Fig. 1f).

### Invasion rate is affected by the presence of flow as well as collagen concentration

**Figure 2:**
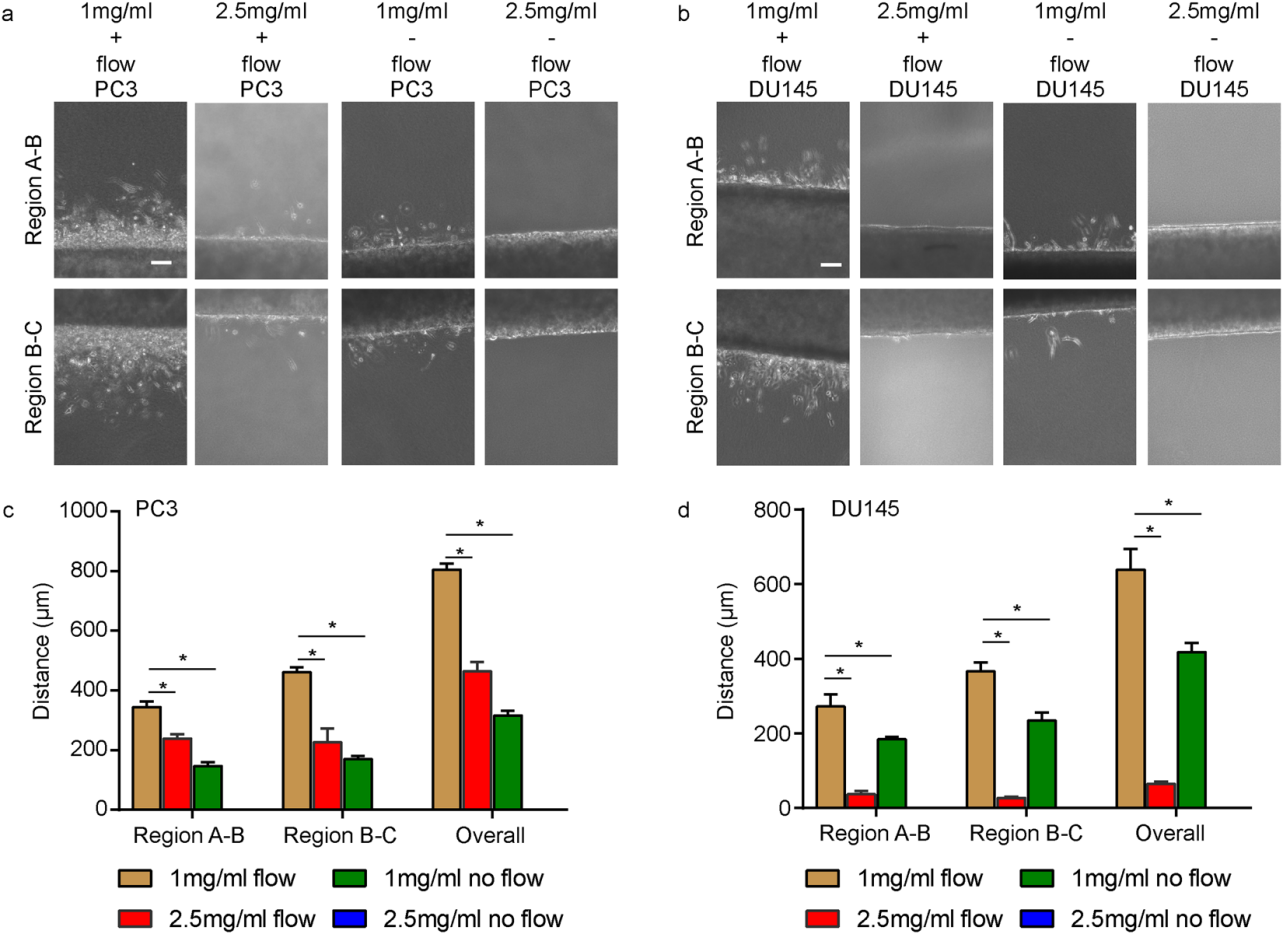
Modulating collagen concentration or flow affects cancer cell invasion. (**a**) Phase images of PC3 cells grown under different conditions of collagen density (1 mg ml^−1^ or 2.5 mg ml^−1^) or flow (+ flow or − flow). Region A-B is shown on the top row while region B-C is on the bottom row. (**b**) Phase images of DU145 cells grown under different conditions of collagen density (1 mg ml^−1^ or 2.5 mg ml^−1^) or flow (+ flow or − flow). Region A-B is shown on the top row while region B-C is on the bottom row. Scale bars = 100 μm. (**c**) Maximum invasion distance of PC3 cells grown in tumoroids under different conditions as indicated. (**d**) Maximum invasion distance of DU145 cells grown in tumoroids under different conditions as indicated. Data were plotted as the mean ± s.e.m, with *n* = 3 biological replicates. *P*-values were calculated using two-tailed unpaired *t*-test with respect to control (1 mg/ml control). *P < 0.05.

The compatibility of the device with low concentrations of collagen even in the presence of flow permitted us to further study how collagen concentration would affect invasion. By increasing collagen concentration in the device by 2.5-fold (from 1 mg/ml to 2.5 mg/ml), the maximum invasion in both regions A-B and B-C was reduced by about two-fold for PC3 tumoroids by one week culture (Fig. 2a,c). However, a similar increase in collagen concentration in DU145 tumoroids resulted in a decrease in maximum invasion in both regions A-B and B-C by about ten-fold (Fig. 2b,d). Furthermore, to examine whether the inclusion of flow and the resulting interstitial pressure and flow in the device would affect invasion, tumoroids grown in both 1 mg/ml collagen were subjected to no flow conditions, with growth medium added manually daily. In PC3 tumoroids, the exclusion of flow in devices with 1 mg/ml of collagen reduced maximum invasion by approximately 2.5-fold, while for DU145 tumoroids, maximum invasion was reduced by 1.5-fold (Fig. 2). The exclusion of flow in devices with 2.5 mg/ml collagen resulted in no observable invasion by one week for either PC3 or DU145 tumoroids (Fig. 2).

### Tumor invasion front and invading cells are more proliferative than tumoroid interior and express higher levels of GDF15

**Figure 3:**
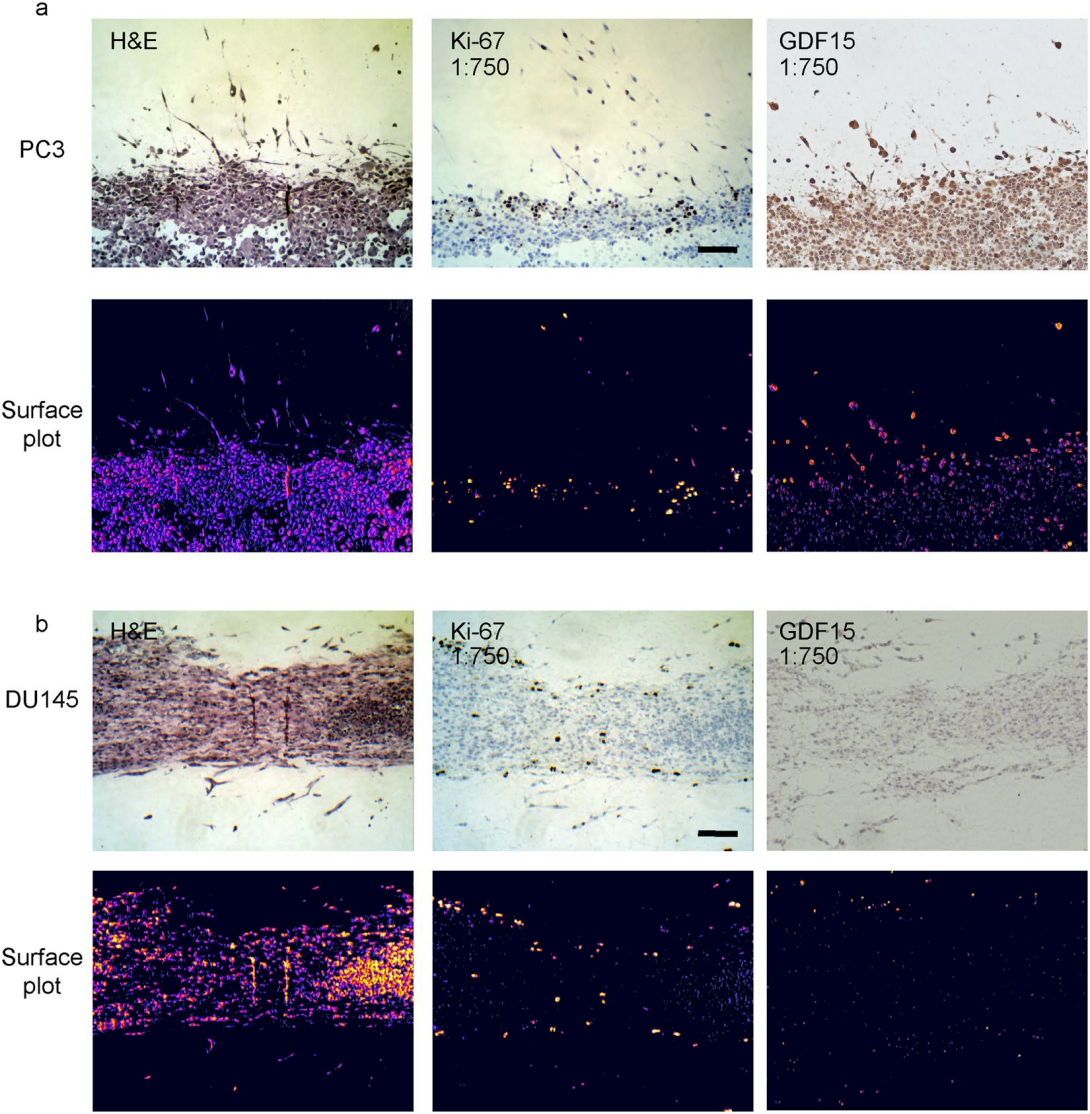
Immunohistochemical staining of tumoroids. PC3 (**a**) and DU145 (**b**) tumoroids were harvested after 3 weeks, fixed, and embedded in paraffin. Sections were stained with H&E (left) or for Ki-67 (middle) and GDF15 (right). 3D surface plot was obtained for H&E, Ki67 and GDF15 staining intensity (bottom). Scale bars = 100 μm.

To examine whether cells located at the invasion front were phenotypically different from the tumoroid interior, we extracted and fixed the tumoroids in 4% paraformaldehyde followed by embedding in paraffin. Sections of the tumoroids were stained with hematoxylin and eosin (H&E) and for Ki-67, a molecular marker of cell proliferation and metastasis^31–33^. Importantly, it has been reported in different patient tumor types that Ki-67 positive cells preferentially locate at the invasion front, suggesting a correlation between Ki-67 expression and invasion^34–36^. H&E staining revealed unique *in situ* features of the tumoroid, such as regions of cellular debris, an indicator of cell death, in the interior of PC3, but not in DU145 tumoroid (Fig. 3a,b). Furthermore, DU145 cells were arranged in layers of cells near the interface between the tumoroid and collagen gel, distinctly different from clusters of round cells in PC3 tumoroid at the same location. We further observed prominent Ki-67 staining mainly localized at the interface between the tumoroid and collagen or in invading cells in both PC3 and DU145 tumoroids (Fig. 3a,b and Supplemental Fig. 3). This observation, which is consistent with clinical findings, again supports a positive correlation between proliferation and active invasion.

To examine whether the fluidic device for long-term 3D tumoroid culture could be used to screen for potential markers of invasion, we harvested and stained tumoroids for growth differentiation factor 15 (GDF15). Elevated serum levels of GDF15 have been found in metastatic prostate cancer patients and is strongly associated with poor clinical outcome^37^. Furthermore, it has been recently demonstrated that GDF15 expression may be regulated by mechanotransduction and that GDF15 expression increases in palisading cancer cells from xenograft samples^37, 38^. Together, these suggest that GDF15 expression could be affected by the environment during invasion and may serve as a marker of active invasion. To examine this possibility, PC3 and DU145 tumoroids were both stained for GDF15, revealing that invading cells did have greater GDF15 expression, which is clearly more evident for PC3 tumoroids (Fig. 3). It should be noted that DU145 cells normally express a lower level of GDF15 than PC3^37, 39^, consistent with a lower GDF15 staining intensity in DU145 tumoroids compared with PC3 tumoroids (Fig. 3). For better visualization of GDF15 expression in DU145 tumoroids, DU145 tumoroids were further re-stained using a 1:100 dilution of GDF15 antibody. Consistently, greater GDF15 expression was observed for invading cells in DU145 tumoroids (Supp. Fig. 3).

## Discussion

In this study, we have successfully designed and constructed a robust and versatile fluidic device for long-term 3D tumoroid culture within a fluidic channel. This device does not require high concentrations of collagen nor additional surface treatment of PDMS to hold the collagen gel or molded channels in place. Instead, the collagen gel was physically secured in place using PTFE adaptors incorporated into the PDMS chamber. This helped the device withstand the effects of fluid flow rates up to 30 μl min^−1^ for prolonged periods of culture time (3 weeks) without structural compromise to the collagen gel^23, 24^. The inclusion of a separate channel without external flow was to serve as a control although it could be potentially used to deliver drugs for screening purposes in the future. While having fluid flow in this system results in multiple variables such as interstitial pressure and flow, we consider the inclusion of these forces more reflective of the *in vivo* environment where the presence of interstitial pressure and flow can affect cell invasion^14–17^. This is further supported by the results of this study that demonstrated that both PC3 and DU145 tumoroids were less invasive in the absence of such forces, supporting the importance of incorporating flow to study invasion. The compatibility of the device with lower concentrations of collagen was also key to tracking the formation of the invasion front with phase contrast microscopy, making this device compatible with patient tumor samples which are typically not genetically modified to express fluorescence markers. Being compatible with low concentrations of collagen further allowed the study how collagen concentration affects invasion. As demonstrated in this study, increasing collagen concentration affected PC3 and DU145 cells differently. While a 2.5-fold increase in collagen concentration resulted in a two-fold decrease in PC3 cell invasion, it decreased invasion in DU145 by approximately ten-fold. Taken together, these results allude to the biological heterogeneity existing between different cells and highlight the utility of this system to screen for biological heterogeneity between invading and non-invading cells.

Excitingly, this 3D tumoroid culture model was able to recapitulate *in situ* features of tumor invasion including the invasion front which can be preserved, processed and stained using conventional immunohistochemistry. This allowed us to further study the heterogeneity existing between the different cell lines PC3 and DU145, including the invasion pattern as well as molecular phenotypes. Distinct features of the invasion front for each cell line were observed, probably reflective of the inherent differences between these two cell lines and highlighting the functionality of using 3D tumoroid culture to characterize tumor invasion. On a molecular level, positive Ki-67 staining in cells located at the invasion front or near the interface between the tumoroid and collagen suggested that invading cells were in a state of active cell proliferation and likely different phenotypically from those located inside the tumoroid. Ki-67 immunolabeling index has been used as an independent predictor of patient outcome for multiple cancer types^32, 40^. A positive correlation between Ki-67 staining and the invasion front in patient tumors has been previously reported^34–36^, although the defined location of a tumor invasion front in patient tumors may be subjective. In this study, our 3D tumoroid culture recapitulated a visually distinct tumor invasion front which further revealed a positive correlation between Ki-67 staining and the invasion front in prostate cancer tumoroids, making this tumoroid culture a suitable *in vitro* preclinical model for cancer development and progression. Moreover, there is a potential for using this device to screen for novel markers of invasion as supported by the results obtained for GDF15. While elevated serum levels of GDF15 has been associated with metastatic prostate cancer and poor clinical outcome, it remains unclear whether GDF15 was associated with early stage invasion. Using our system for PC3 and DU145 cells, we clearly demonstrate that GDF15 expression was higher in invading cells than those inside the tumoroid, suggesting an association between GDF15 expression and invasion. This preliminary finding reinforces the phenotypic differences between invading and non-invading cells seen with the Ki-67 staining. It further opens up new avenues of research that would allow future study of the causal relationship between GDF15 and invasion.

It remains a future goal to fully characterize the molecular phenotype as well as study the dynamics of phenotypic plasticity of invading leader cells at the tumor invasion front using this 3D tumoroid culture system. Future work can also be directed toward using this 3D tumoroid culture system to characterize primary tumors, biopsies or cell lines derived from cancer patients to facilitate risk stratification of cancer patients and develop personalized treatment strategies.

## Methods

### Tissue Culture

PC3 and DU145 cell lines were purchased from American Type Culture Collection and cultured using 1× RPMI growth medium (Life Technologies) supplemented with 10% fetal bovine serum (Life Technologies) and 100 μg/ml penicillin/streptomycin (Life Technologies). The cell lines were authenticated by the provider using short tandem repeat analysis, and a fresh batch of cells was used after 6 months of culture. For subculture, cells were first rinsed with 1× PBS followed by addition of trypsin and incubation at 37 °C for 5 min. Cells were re-suspended in growth medium and centrifuged at 300 g for 5 min. After centrifugation, excess growth medium was aspirated, and the cell pellet was re-suspended in growth medium. Re-suspended cells were seeded in a 75 cm^2^ tissue culture flask (Greiner) and placed in a 37 °C incubator supplemented with 5% CO_2_. Growth medium was changed every 2 days, and cells were subcultured every 3–4 days.

### Fluidic Tumoroid Culture

The functional fluidic device consisted of three fluidic channels molded in a 1 mg/ml collagen I hydrogel. We first constructed a sealed PDMS chamber with three channels running parallel through. To this end, three 23G needle syringes (BD) were first suspended 5 mm above a 150 mm plastic dish cover (BD) using pieces of acrylic as supports. PDMS prepolymer (with a 1:10 ratio of crosslinker to PDMS base) was then poured into the dish cover till the dish cover was nearly full. PDMS inside the dish cover was cured at 60 °C overnight. After curing, the 23 G needles were extracted, leaving behind 3 channels suspended in a PDMS block. A chamber of 2 cm × 2 cm was then cut into the PDMS block using a surgical blade, creating a chamber with openings for three channels running through the middle of the chamber. On one side of the chamber parallel to the channels, an additional 5 mm semi-circle punch was made in the chamber to act as an air outlet when introducing collagen in latter steps. At each of the channel openings, we placed a piece of polytetrafluorethylene (PTFE) tubing (#24 AWG thin wall tubing; Cole-Palmer) to serve as an adaptor. The PTFE adaptors ensured that the fluidic channels were held in place within the collagen gel and that fluid flow or cell seeding was directed into the channel throughout the experiment. The middle channel adaptor was placed 5 mm apart from the side channel adaptors to ensure that cancer cells seeded were not subject to any drastic change in fluid pressure as fluid initially flowed into the channel (Fig. 1a, left, Supplementary Fig. 2). The final device was constructed by sealing the chamber with a microscope slide (Fisher Scientific) at the base and a 1mm thick PDMS layer at the top using plasma bonding (Harrick Plasma). To form the fluidic channels in collagen I hydrogel, a 25 G needle (BD) was first placed through each channel opening. Before injecting 1 mg/ml collagen I (Corning) into the device, an additional 23 G needle was inserted into the air outlet described earlier to allow air from the chamber to escape during collagen gel injection. Collagen gel was allowed to polymerize at 37 °C for 30 min, after which the 25 G syringe needles were extracted, leaving a molded 500 μm diameter fluidic channel within the collagen gel.

1 × 10^6^ cancer cells were introduced into the middle channel, thereby confining the population of cells at the same site within the device and forming a tumor mass 500 μm in diameter. Both ends of the middle channel were sealed using sealing wax. A peristaltic pump (Thermo Fisher) was used to deliver 3 ml growth medium at a rate of 30 μl/min into channel A in a circulatory manner. To contain the growth medium, a PDMS chamber with a single channel running through was constructed to act as a reservoir for growth medium. Growth medium in the reservoir was changed every 2 days to ensure fresh medium was provided to the tumoroid.

Manual feeding of the devices was achieved by removing the PDMS layer on top of the device after cell seeding and adding growth medium onto the collagen gel. Growth medium was changed daily by aspiration.

### Invasion Distance Measurement

Mosaic images of the tumoroid were created using the Zeiss AxioVision Observer (Zeiss). Images were taken using a 10× objective. The maximum invasion distance of tumoroid was calculated as the vertical distance between the furthest observable cancer cell or edge of invasion front from either side of the middle channel wall along the entire tumoroid. Statistical analysis was performed using student’s t-test (GraphPad Prism).

### Immunohistochemical Staining

The PDMS devices were first cut open to harvest the tumoroids. The tumoroids were placed in 4% paraformaldehyde fixative for 3 days to ensure complete fixing. The tumoroids were then embedded, sectioned, and stained by the Unit for Laboratory Animal Medicine *In-vivo* Animal Core at the University of Michigan. Hematoxylin and eosin (H&E) staining was performed and immunohistochemical (IHC) staining for Ki-67 was conducted with hematoxylin counterstain. GDF15 (Santa Cruz) immunohistochemical staining was performed at a 1:750 dilution and 1:100. 3D surface plot of IHC slides was performed using ImageJ (NIH).

### Simulation of Fluid Pressure

Simulation of the pressure field within the collagen gel was performed using the Free and Porous Media Model in COMSOL Multiphysics. A stationary 3D model was generated using the following parameters: 1. Channel diameter of 500 μm for all three circular channels; 2. Channel A had a uniform inlet flow velocity of 0.02 m/s with a zero-pressure outlet, while Channel B and Channel C were filled with fluid and their inlets and outlets were all closed; 3. Based on a previous report, the permeability and porosity of the collagen gel were set to be 10^−14^ m^2^ and 0.998, respectively^41^; 4. The growth medium density was 980 kg m^−3^, with a dynamic viscosity of 10^−3^ Pa **·** s.

### Data availability

All data generated or analyzed during this study are included in this published article (and its Supplementary Information files).

## Electronic supplementary material

Supplementary information
