# Supplementary material for: Tracking the tumor invasion front using long-term fluidic tumoroid culture
Source: Sci Rep. 2017 Sep 7;7:10784. doi: 10.1038/s41598-017-10874-1 (PMC5589910; doi:10.1038/s41598-017-10874-1)
Supplement: Supplementary file 1 — Supplementary information [file 41598_2017_10874_MOESM1_ESM.doc]

**Supplementary Information**

**Tracking the tumor invasion front using**

**long-term fluidic tumoroid culture**

Koh Meng Aw Yong^1,#^, Zida Li^1^, Sofia D. Merajver^3^ and Jianping Fu^1,2,4,*^

^1^Department of Mechanical Engineering, University of Michigan, Ann Arbor, MI 48109, USA; ^2^Department of Biomedical Engineering, University of Michigan, Ann Arbor, MI 48109, USA;

^3^ Department of Internal Hematology and Oncology, University of Michigan, Ann Arbor MI 48109, USA;

^4^Department of Cell and Developmental Biology, University of Michigan Medical School, Ann Arbor, MI 48109, USA.

**Disclosure**

The authors declare that there are no conflicts of interest.

^#^Current address: Department of Internal Medicine, Hematology, and Oncology, University of Michigan Medical School, Ann Arbor, MI 48109, USA.

*Corresponding author ([jpfu@umich.edu](mailto:jpfu@umich.edu)).

**SUPPLEMENTARY FIGURES AND CAPTIONS**

**
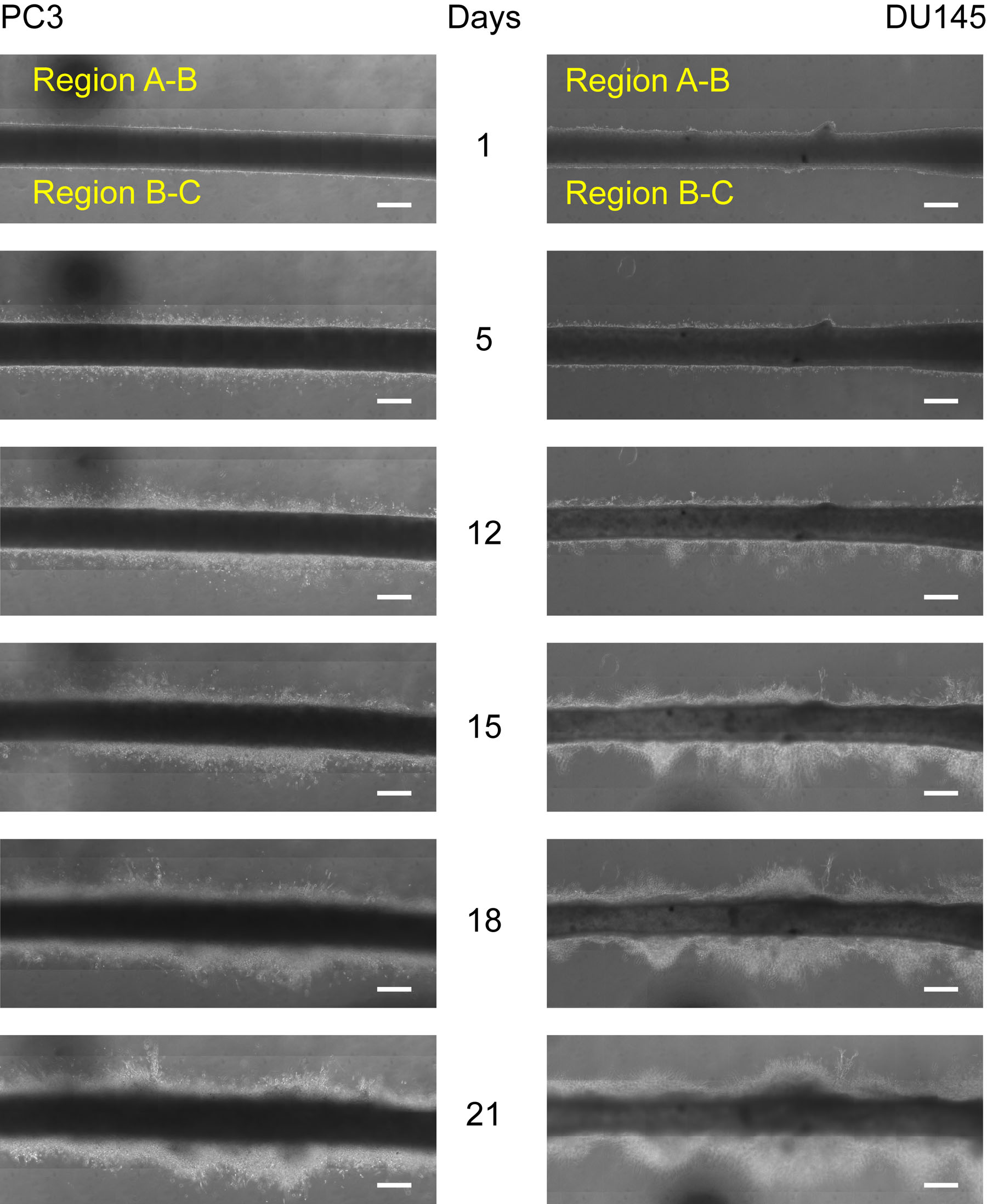
**

**Supplementary Figure 1. Development of the invasion front in cultured tumoroids.** Mosaic phase images of the entire tumoroid were taken over time to demonstrate the formation of the invasion front into regions A-B and B-C. Spatiotemporal analysis of PC3 (left) and DU145 (right) tumoroids shows the formation of multiple invasion fronts from the tumoroid. Scale bars, 500 μm.


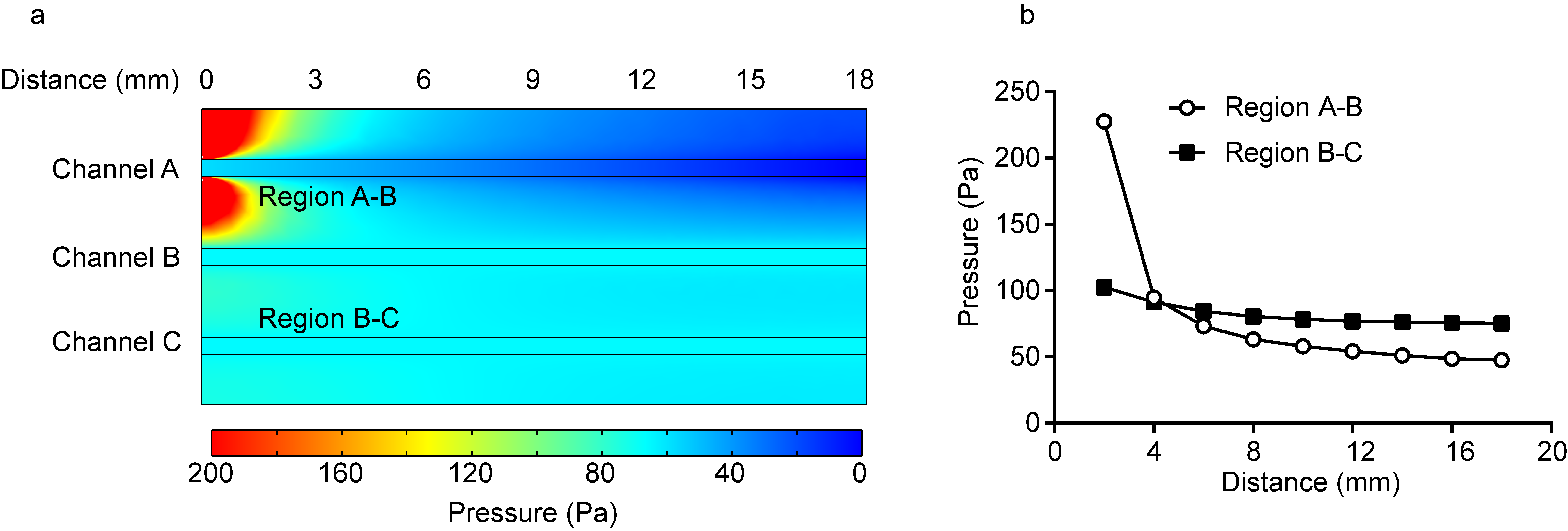


**Supplementary Figure 2. Heat map showing hydrostatic pressure within the fluidic device.** (a) Heat map revealed an initial region in region A-B with elevated pressure near the opening of channel A. Pressure within region A-B decreased rapidly as fluid flowed through channel A. The overall pressure within region B-C was higher than in region A-B. (b) Plot of average pressure values distribution in both regions throughout the length of the device.


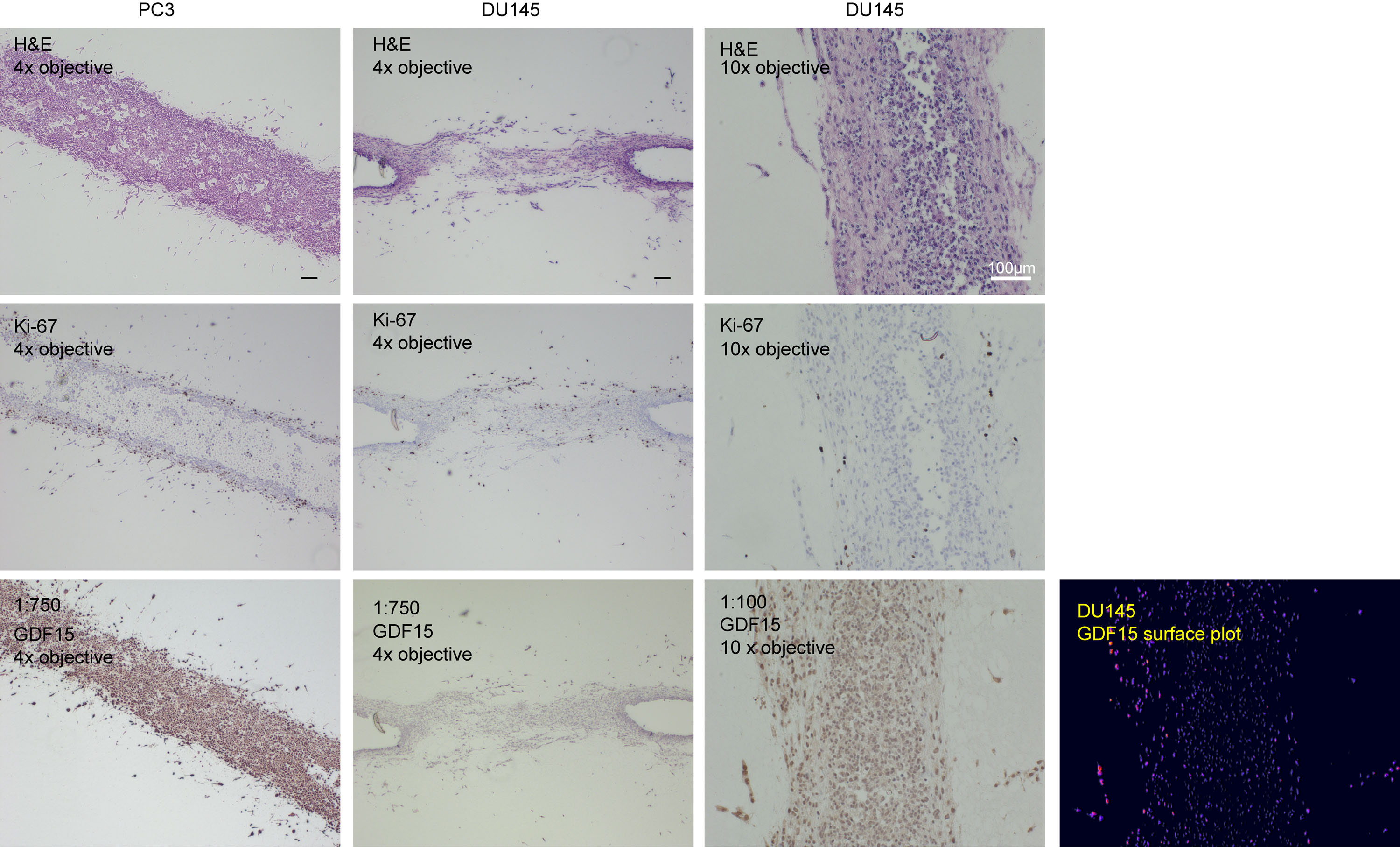


**Supplementary Figure 3. Low power magnification of PC3 and DU145 tumoroids.** H&E, Ki-67 and GDF15 IHC images were taken using 4x objective. PC3 tumoroids are shown on the left-most column and DU145 on the right three columns. H&E (top row), Ki-67 (middle row) and GDF15 (bottom row). Ki-67 and GDF15 staining were observed to be stronger at the tumoroid interface and invading cells in PC3 and DU145 tumoroids. Scale bars=100μm.
